# Patterns of physical activity in different domains and implications for intervention in a multi-ethnic Asian population: a cross-sectional study

**DOI:** 10.1186/1471-2458-10-644

**Published:** 2010-10-25

**Authors:** Ei Ei Khaing Nang, Eric YH Khoo, Agus Salim, E Shyong Tai, Jeannette Lee, Rob M Van Dam

**Affiliations:** 1 Department of Epidemiology and Public Health, Yong Loo Lin School of Medicine, National University of Singapore, Singapore, Republic of Singapore; 2 Department of Medicine, Yong Loo Lin School of Medicine, National University of Singapore, Singapore, Republic of Singapore; 3 Department of Nutrition, Harvard School of Public Health, Boston MA, USA

## Abstract

**Background:**

The benefits of regular physical activity for quality of life and disease prevention have been well documented. Identification of low activity groups would facilitate interventional programs. Many studies have focussed on leisure time activity, which may not capture the spectrum of physical activity relevant to disease prevention. Furthermore, few studies have been conducted in urban Asian settings.

**Methods:**

We evaluated physical activity in different domains (leisure time, occupational, household and transportation) and its sociodemographic determinants in 4750 adult Chinese, Malay, and Asian Indian Singaporeans. Physical activity was assessed using locally validated questionnaires.

**Results:**

Occupational and household activity contributed substantially more to total physical activity than leisure time or transportation activity. However, when only activity of at least moderate intensity was considered leisure time activity contributed most to total physical activity. Higher socio-economic status was associated with more leisure time activity, but less total physical activity due to reduced activity in the other domains. Chinese ethnicity was also associated with less total physical activity as a result of less activity in non-leisure time domains.

**Conclusions:**

In assessing levels of physical activity and recommending changes, it is important to consider physical activity in different domains. Focus on leisure-time physical activity alone could identify the wrong groups for intervention and miss opportunities for increasing physical activity in populations.

## Background

It is widely recognized that regular physical activity has many health benefits including a reduction in risk of cardiovascular diseases, type 2 diabetes, hypertension, certain cancers, osteoporosis, dyslipidaemia, anxiety and depression [1,2]. Public health programmes have therefore emphasized increases in physical activity and commonly used guidelines recommend that all adults accumulate at least 30 minutes per day of activity of at least moderate intensity (equivalent to walking at 3 to 4 miles per hour) [3].

Recent studies have emphasized the value of assessing total physical activity (leisure time, occupational, transportation and household) rather than focussing on one domain or aspect of physical activity, especially in certain groups of individuals such as women, ethnic minorities or those with lower social economic status [4,5]. In addition, Westerterp demonstrated that vigorous activities such as sports may not contribute as much to total energy expenditure as moderate-intensity activities because vigorous activities are often of short duration [6]. Furthermore, there is also evidence that in the general population, regular light-to-moderate activities such as walking may be beneficial for lowering risk of coronary heart disease and stroke [7-10]. In a U.S. cohort study, women who were walking regularly even with light intensity (walking pace less than 2.0 mph) had a lower risk of coronary heart disease than women who did not walk regularly [7]. As a result, light-to-moderate-intensity everyday activities may be highly relevant for promoting physical activity in the general population. Thus, a broader approach to the assessment of energy expenditure associated with physical activity will more reliably identify groups with the lowest total physical activity which could have greater priority for public health interventions.

Most previous studies have been conducted in western populations [11-13], and few have evaluated different domains of physical activity in diverse populations in an urban Asian setting [14,15]. We therefore evaluated the characteristics of individuals participating in activities in different domains in a population-based study of Chinese, Malay, and Asian Indian in Singapore. These data can assist in identifying the domains of activity that can be targeted and the low physical activity groups who may benefit most from public health intervention.

## Methods

### Study population

In this study, conducted between 2004 and 2007, 10,080 subjects who had previously participated in population-based cross-sectional surveys in Singapore between 1982 and 1998 were invited. These previous surveys were all conducted in random sample of individuals from the Singapore population, with disproportionate sampling stratified by ethnicity to increase the numbers for ethnic minority groups (Malays and Asian Indians) [16]. Participants who were deceased at time of follow-up (through data-linkage with the Registry of Births and Deaths) were excluded from the study (n = 559). Six participants who had emigrated and 102 participants, who had error in identity card number and couldn't follow up, were excluded.

A questionnaire was administered by investigators at the participant's home. Questionnaires were in English and when needed interviewers provided additional explanation in Chinese, Malay or Tamil. Three home visits on three different occasions including one weekend and weekday were made before a participant was deemed non-contactable. 2306 participants were non-contactable. Of the remaining participants, thirty (0.3%) refused to take part. All the interviewed participants were subsequently invited to attend a health examination for additional tests and collection of blood samples shortly after the home visit. A total of 7,744 (76.8% response rate) were interviewed, of which 5,164 (66.7% response rate, or 51.3% of total eligible participants) participants attended the health examination. Of note a high percentage 37.8% of the study population declined to reveal their household income. Ethics approval was obtained from the Institutional Review Boards of National University of Singapore and Singapore General Hospital. Informed consent was obtained from all participants before conduct of study.

### Assessment of Physical activity

Physical activity was assessed by interviewer-administered questionnaire with a recall period of the previous 3 months. The questionnaire was adapted from several established questionnaires validated in other populations [17-19] and encompassed transportation, occupation, leisure time and household activities. Participants were asked the type and duration of various activities. Then a metabolic equivalent of task (MET) value was assigned to each reported activity according to the compendium by Ainsworth et al [20]. One MET unit is defined as the energy expenditure for sitting quietly, which for the average adult is approximately 3.5 ml of oxygen × kg bodyweight^-1 ^× min^-1 ^or 1 kcal × kg body weight^-1 ^× h^-1 ^[20]. The minutes were converted to hours and weekly energy expenditure from physical activity (Kcal/week) was computed as follows: hours spent on activity per day × numbers of day per week × METs × body weight in kg [21,22]. All light, moderate, and vigorous activities were included in this calculation. For example, if a 60-kg participant reported bowling activity for 60 minutes per day for 3 times a week, then his energy expenditure for this activity was calculated as 1 hr × 3 days × 3 METs × 60 kg resulting in 540 Kcal/week.

The questions on transportation activity were adapted from National Health Survey 2004 questionnaire [23] which asked about walking or cycling for transport for at least 10 minutes. The duration, frequency and the intensity of the activity (light, moderate, or vigorous) were recorded. Questions on occupational activity were based on the validated Modifiable Activity Questionnaire [18,24]. Participants were asked to list all jobs held during the past 3 months. For each job entry, data was collected for the job schedule and job activity was determined by the number of hours spent sitting at work and the most common physical activities performed when not sitting. Leisure time activity was adapted from the Minnesota leisure time activity questionnaire covering a total of 48 specific activities and open questions about possible other activities which has been validated in various populations [19,25-27]. For each activity, participants identified the frequency and the average duration of participation in each activity. Household activity was adapted from the Yale physical activity questionnaire which covers housework, yard work and caretaking for elderly persons or children and has been validated in diverse populations [17,28,29]. Participants were asked about the type of activity performed and the frequency and duration of each activity.

We validated the combined physical activity questionnaire using Actical accelerometers in Singapore population. The Actical accelerometer has been validated previously showing good reliability and accuracy for estimating the time spent in moderate and vigorous physical activity [30,31] and has been widely used in epidemiological studies [32]. A convenience sample of 120 Singaporean adults, aged 21-80 years (49 Chinese, 31 Malays and 40 Asian Indians; 54 males and 66 females) was recruited. Participants were given instruction on wearing the Actical accelerometer. The participants were required to wear this device for five consecutive days (3 weekdays and 2 weekend days) during all waking hours (except during water-based activities). At the end of the monitoring period, the participants completed the same interviewer-administered physical activity questionnaire as was used in the main study. The correlation between the accelerometer and the physical activity questionnaire estimates were 0.19 (p = 0.03) for moderate (3 to 6 METs) and 0.40 (p < 0.001) for vigorous (>6 METs) activity.

### Classification of physical activity

"Have no activity" was defined as a person not participating in any physical activity and "Have any activity" was defined as a person participating in any amount of physical activity in the respective domain i.e. any amount of light, moderate or vigorous activity. The recommended level of physical activity was defined as at least 150 minutes a week of moderately-intense or 75 minutes a week of vigorously-intense aerobic physical activity, or an equivalent combination of moderate and vigorous intensity aerobic activity [3]. Moderate intensity was defined as 3 to 6 metabolic equivalents (METs) and vigorous intensity was defined as more than 6 METs [3].

### Assessment of socio demographic factors

Baseline information about socio demographic factors (age, gender, ethnicity, education, work status, household income in Singapore $ per month and type of housing) was recorded by interviewer-administered questionnaire. Education was determined as the highest level achieved. This was divided into 4 categories: no education/primary, secondary, technical school and university.

### Statistical Analysis

5164 participants attend the health examination, of which 143 were excluded due to missing data on weight. In addition, to exclude outliers, the ratio of the energy intake to energy expenditure was calculated and 271 participants, who were in the lowest 2.5 percentile and in the highest 2.5 percentiles, were excluded. A total of 4750 participants were left for analysis. For occupational activity, only the 3267 participants who were employed were included in the analysis.

All statistical analyses were performed using Stata 10 for Windows (Stata Corporation, College station, Texas, USA). The participants were divided into two groups; "have no activity" and "have any activity" and chi-square tests were used to test whether there was an association between participation in physical activity and various sociodemographic factors. For "have any activity" group, the energy expenditure was reported in median and as the distribution of energy expenditure in each domain was skewed, the Kruskal Wallis test was therefore used to assess the association between level of energy expenditure and sociodemographic variables.

**Figure 1:**
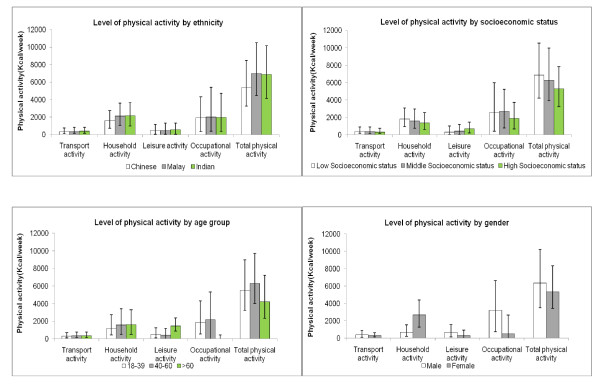
**Level of physical activity according to sociodemographic characteristics**. Physical activity includes light, moderate and vigorous activity. The activity in each domain, including occupational activity, is based on all eligible participants (N = 4750) i.e. both employed and unemployed participants. a. Median (interquartile range) level of physical activity by ethnicity, adjusted for age and gender. b. Median (interquartile range) level of physical activity by socioeconomic status, adjusted for age and gender. c. Median (interquartile range) level of physical activity by age group. d. Median (interquartile range) level of physical activity by gender.

**Figure 2:**
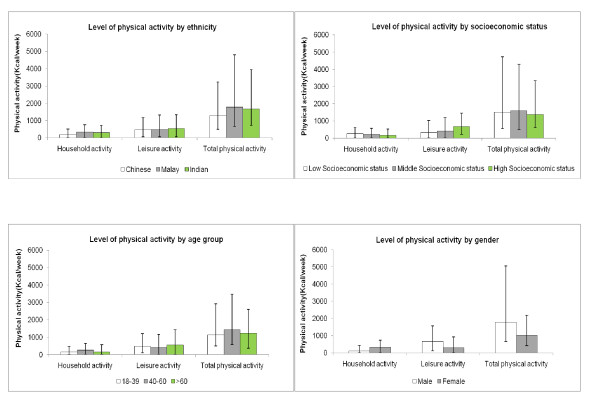
**Level of physical activity of at least moderate intensity according to sociodemographic characteristics**. The activity in each domain was based on all eligible participants (N = 4750). Transportation and occupational activity were not shown because less than 25% participated in any activities of at least moderate intensity in these domains. However, total activity does include activity from all four domains. a. Median (interquartile range) level of physical activity of at least moderate intensity by ethnicity, adjusted for age and gender. b. Median (interquartile range) level of physical activity of at least moderate intensity by socioenocomic status, adjusted for age and gender. c. Median (interquartile range) level of physical activity of at least moderate intensity by age group. d. Median (interquartile range) level of physical activity of at least moderate intensity by gender.

Age-and gender adjusted median, 25^th ^and 75th percentiles of the activity level of the whole population (i.e includes both no activity and any activity group) were obtained by median regression and presented in the figures stratified by ethnicity and socioeconomic status (SES). Crude median, 25^th ^and 75^th ^percentiles of activity level stratified by age and gender are also shown in the figures. The amount of physical activity based on activity at all intensities (i.e. light, moderate and vigorous) is shown in figure 1, whereas the amount of physical activity based only activity of at least moderate intensity is shown in figure 2. Less than 25% of the population had any transportation or occupational activity of at least moderate intensity. Therefore, these two domains were not presented in figure 2. However, the data for total physical activity level in this figure still covers all 4 domains. For occupational activity, the analysis for tables included only those participants who were currently working while the figures included both working and non working group to be consistent with other domains in the figures.

SES was expressed by individual indicators such as highest level of education, work status, household income and type of housing in univariate and multivariate analysis in the tables. However, for figure 1 &2b, a single SES index was developed from education, household income and type of housing. Participants who had missing answer on any of these indicators were excluded (1 missing answer for education and 1794 declined to answer household income). A total of 1794 participants were excluded and 2956 participants were left for SES index analysis. SES index from these three indicators was obtained by performing principal component analysis and using the first and second tertiles of the first principal component score to classify subjects into low, middle and high SES. All statistical tests were two-sided with a level of significance defined as a *P*-value < 0.05.

## Results

Population characteristics stratified by gender and ethnic group are shown in Table 1. Participants were of Chinese (67.6%), Malay (16.9%), and Asian Indian (15.4%) ethnicity. More than 80% of men in each ethnic group were employed as compared with 61.5%, 45.0% and 48.0% of Chinese, Malay and Indian women respectively. Approximately 14% of men and 4% of women were retired. Chinese participants were more likely to have a university education, a higher income and live in private housing as compared with other ethnic groups (Table 1).

**Table 1:** Socio-demographic characteristics of study population

	Male (N = 2280)			Female (N = 2470)		
	Chinese N = 1530	Malay N = 392	Indian N = 356	Chinese N = 1682	Malay N = 411	Indian N = 375
**Characteristics**	**%**	**%**	**%**	**%**	**%**	**%**
**Age group**						
18-39 years	20.5	13.5	11.5	22.8	19.5	13.9
40-60 years	58.6	68.6	65.7	60.3	65.2	69.1
>60 years	20.9	17.9	22.8	16.9	15.3	17.1
**Highest level of education**						
None/primary	20.9	24.7	17.1	27.8	36.0	37.6
Secondary	33.0	48.5	42.4	37.6	46.7	41.1
Technical school	23.0	23.5	23.9	19.4	15.8	11.5
University	23.1	3.3	16.6	15.2	1.5	9.9
**Work status**						
Working	80.8	82.4	82.6	61.5	45.0	48.0
Student	0.6	0.5	0.3	0.3	0.5	0.3
Homemaker	0.1	0	0.3	31.0	48.2	44.8
Retired	14.5	13.8	13.5	4.9	2.9	4.3
Unemployed (able to work)	2.4	1.8	2.0	1.4	2.0	1.6
Unemployed (unable to work)	1.1	1.0	0.8	0.4	0.2	0.3
#Others	0.7	0.5	0.6	0.6	1.2	0.8
**Household income (S$/month)**						
Less than $2000	14.5	31.1	24.4	14.5	23.4	26.7
$2000 to $3999	20.1	21.7	23.3	16.9	24.6	21.6
$4000 to $5999	12.8	8.9	11.5	10.6	5.6	8.0
$6000 to $9999	10.2	2.8	9.8	10.6	3.2	5.1
More than $ 10 000	7.1	1.0	4.8	5.2	0.5	1.6
Decline to answer	35.3	34.4	26.1	42.1	42.8	37.1
**Type of housing**						
1-2 room flat	1.1	2.3	0.8	1.0	3.9	2.9
3 room flat	13.7	15.8	12.1	16.2	15.3	14.4
4 room flat	32.2	45.7	37.9	31.3	43.3	38.1
5 room or executive flat	31.0	29.1	36.0	29.1	32.4	33.3
Private condominium	12.4	3.3	4.5	12.0	2.9	4.3
Private house (landed property)	9.7	3.8	8.7	10.5	2.2	6.9

#Others: disabled persons, persons with large private financial means, prisoners, patients of mental hospitals, inhabitants of homes for the aged as well as those who were awaiting call-up for national military service are included in this category.

### Physical activity by domain

Household and occupational activity contributed most to total physical activity, whereas leisure and transportation activity contributed much less (Figure 1). However, when only activity of at least moderate intensity was considered, leisure time activity contributed most to physical activity (Figure 2).

Leisure-time activity was higher in men, in the older age group, and those with higher SES. No substantial differences were observed by ethnicity (Figure 1 &2). In addition, we considered the proportion participating in any leisure time activity and the level of activity among those that participated in leisure time activity separately (Table 2). 73% reported participating in at least some leisure time activity. Men were both more likely to participate in leisure time activity and to have a higher level of activity. Similarly, students and retired persons had both a higher participation in leisure time activity and a higher level of activity than the employed and homemakers. However, older persons were less likely to participate in leisure time activity than younger persons, but had a higher level of activity if they participated. A higher SES was associated with a higher likelihood of participating in leisure time activity, rather than a higher level of activity for those who participated. Associations for different measures of SES (education, income, type of housing) were similar. When only leisure time activity of at least moderate intensity was considered, associations with sociodemographic characteristics were generally similar (Figure 2). However, the association between older age and more leisure time activity largely disappeared.

**Table 2:** Leisure time and occupational physical activity according to sociodemographic characteristics.

	Leisure time activity		Occupational activity*	
	Any activity	Median†	Any activity	Median†
	N (%)	(Kcal/week)	N (%)	(Kcal/week)
	**3449 (73)**	**801(360-1600)**	**3122(96)**	**3527(1534-6298)**
**Age group**				
18-39 years	738 (80)	706 (341-1493)	751(95)	2600(1134-4957)
40-60 year	2085 (71)	799 (350-1595)	2147(97)	3861(1682-6633)
>60 years	626 (71)	966 (470-1778)	224(93)	4174(2144-7115)
p value	<0.0001	<0.0001	0.06	0.0001
**Gender**				
Male	1779 (78.)	964 (455-1867)	1797(97)	4492(2192-7806)
Female	1669 (68)	648 (293-1308)	1324(94)	2377(991-4561)
p value	<0.0001	<0.0001	<0.0001	0.0001
**Ethnicity**				
Chinese	2327 (73)	777 (359-1530)	2169(96)	3107(1356-5583)
Malay	575 (72)	859 (359-1767)	493(96)	4986(2388-8253)
Asian Indian	546 (75)	907 (365-1676)	457(96)	4385(2034-7098)
p value	0.3	0.02	0.09	0.0001
**Highest level of education**				
None/primary	759 (62)	776 (339-1588)	552(97)	5290(3250-8595)
Secondary	1297 (71)	784 (342-1590)	1224(96)	3919(1608-6938)
Technical school	777 (81)	830 (384-1667)	748(95)	3146(1445-5832)
University	616 (85)	840 (412-1565)	597(94)	2065(1084-3744)
p value	<0.0001	0.4	0.09	0.0001
**Work status**				
Working	2354 (72)	788 (346-1563)	NA	NA
Student	16 (80)	1089 (649-1515)		
Homemaker	620 (70)	757 (354-1499)		
Retired	345 (80)	1148 (528-2080)		
Unemployed (able to work)	65 (74)	932 (501-1685)		
Unemployed (unable to work)	20 (63)	1180 (427-1722)		
#Others	26 (81)	475 (176-2063)		
p value	0.006	<0.0001		
**Household income(S$/month)**				
Less than $2000	552 (64)	739 (363-1630)	566(96)	4750(2344-8000)
$2000 to $3999	663 (70)	763 (327-1534)	707(94)	3990(1788-6930)
$4000 to $5999	405 (80)	794 (354-1504)	401(95)	2772(1303-5126)
$6000 to $9999	332 (80)	800 (409-1713)	342(97)	2204(1022-3965)
More than $ 10 000	196 (87)	933 (443-1773)	184(94)	2123(1195-4029)
Decline to answer	1300 (73)	834 (368-1586)	922(96)	3939(1663-6400)
p value	<0.0001	0.07	0.6	0.0001
**Type of housing**				
1-2 room flat	42 (58)	1196 (568-1924)	34(94)	4352(3216-6707)
3 room flat	460 (65)	767 (332-1538)	430(96)	4341(1833-7236)
4 room flat	1174 (71)	750 (333-1558)	1121(96)	4128(1933-7140)
5 room or executive flat	1080 (74)	801 (350-1574)	1005(95)	3269(1462-5856)
Private condominium	364 (81)	946 (437-1884)	296(95)	2226(1102-4485)
Private house (landed property)	329 (81)	895 (500-1717)	236(95)	2186(1032-4721)
p value	<0.0001	0.0002	0.9	0.0001

Both the proportion of participants participating in an activity and the level of activity among those that engage in that activity is provided.

#Others: disabled persons, persons with large private financial means, prisoners, patients of mental hospitals, inhabitants of homes for the aged and men awaiting call-up for military service are included in this category.

*Occupational activity was only included 3267 participants who were employed at the time of interview. †Median among those with any amount of activity (inter-quartile range). NA: not applicable.

Occupational activity was higher in the middle age group, men, those with low SES and those of Malay ethnicity (Figure1). Having any light, moderate or vigorous occupational activity was reported by 96% of the working population. The percentage having any occupational activity was highest in the middle age group. However, the level of occupational activity was highest in the older age group. Men, Malays, and those with a lower SES had both the highest participation in occupational activity and the highest level of occupational activity (Table 2). The percentage of having moderate activity and above was reported by 25.0% of working population (i.e. 17.2% of the total population) (Data not shown). Transportation activity was similar in the different age and ethnic groups, but was lower for those with a higher SES (Figure 1). 82% of the population participated in at least some transportation activity. Women were more likely to participate in transport activity, but among those that participated in transport activity the level was lower for women than for men (Table 3). Most transportation activity was of light intensity and transportation activity of at least moderate intensity was only reported by 18.0% of the participants.

**Table 3:** Physical activity in the transportation and household domain according to sociodemographic characteristics.

	Transportation activity		Household activity	
	Any activity	**Median**†	Any activity	**Median**†
	N (%)	(Kcal/week)	N (%)	(Kcal/week)
	**3912 (82)**	431 (242-905)	**4346 (92)**	1710 (696-3474)
**Age group**				
18-39 years	729 (79)	421 (246-864)	846 (92)	1322 (571-2983)
40-60 years	2434 (83)	438 (240-917)	2714 (92)	1771 (717-3630)
>60 years	749 (85)	410 (240-876)	786 (89)	1892 (772-3481)
p value	0.003	0.6	0.01	<0.0001
**Gender**				
Male	1807 (79)	507 (291-1061)	1932 (85)	892 (431-1786)
Female	2104 (85)	364 (210-749)	2413 (98)	2765 (1364-4483)
p value	<0.0001	<0.0001	<0.0001	<0.0001
**Ethnicity**				
Chinese	2615 (81)	414 (234-887)	2929 (91)	1507 (619-3095)
Malay	674 (84)	448 (265-958)	744 (93)	2191 (942-4220)
Asian Indian	621 (85)	457 (257-931)	670 (92)	2345 (862-4445)
p value	0.04	0.02	0.5	<0.0001
**Highest level of education**				
None/primary	1066 (86.)	420 (242-945)	1121 (90)	2384 (1058-4039)
Secondary	1542 (85)	445 (253-925)	1686 (92)	1899 (754-3718)
Technical school/diploma	774 (80)	419 (226-890)	883 (92)	1374 (625-2879)
University	529 (73)	418 (219-785)	656 (91)	1038 (418-2183)
p value	<0.0001	0.02	0.3	<0.0001
**Work status**				
Working	2647 (81)	441 (250-318)	2930 (90)	1226 (536-2498)
Student	16 (80)	421 (254-595)	18 (90)	914 (433-1516)
Homemaker/housewife	762 (86)	385 (210-827)	876 (99)	4018 (2777-5806)
Retired	364 (84)	406 (243-871)	386 (89)	1716 (745-3259)
Unemployed(able to work)	76 (86)	531 (316-1164)	83 (94)	1831 (886-3556)
Unemployed(unable to work)	22 (69)	424 (169-1544)	22 (69)	1213 (597-2154)
#Others	22 (7)	650 (357-837)	29 (91)	1537 (830-3782)
p value	0.004	0.01	<0.0001	<0.0001
**Household income(S$/month)**				
Less than $2000	765 (88)	457 (262-1082)	805 (93)	1892 (779-3606)
$2000 to $3999	828 (88)	475 (253-1023)	877 (93)	1621 (704-3474)
$4000 to $5999	413 (82)	477 (238-958)	464 (92)	1489 (661-3267)
$6000 to $9999	318 (77)	426 (234-865)	393 (95)	1284 (583-2591)
More than $ 10 000	149 (66)	388 (204-864)	212 (94)	1023 (389-2285)
Decline to answer	1438 (80)	379 (232-769)	1594 (89)	2009 (754-3775)
p value	<0.0001	0.02	<0.0001	0.001
**Type of housing**				
1-2 room flat	57 (79)	510 (324-869)	66 (92)	2191 (793-3481)
3 room flat	611 (87)	388 (219-837)	645 (92)	1784 (699-3459)
4 room flat	1408 (85)	449 (261-940)	1505 (91)	1848 (771-3606)
5 room or executive flat	1210 (83)	445 (246-903)	1354 (92)	1666 (706-3488)
Private condominium	332 (74)	374 (215-860)	413 (92)	1354 (494-2860)
Private house(landed property)	294 (73)	387 (210-903)	363 (90)	1392 (554-3505)
p value	<0.0001	0.003	0.5	<0.0001

Both the proportion of participants participating in an activity and the level of activity among those that engage in that activity is provided.

#Others: disabled persons, persons with large private financial means, prisoners, patients of mental hospitals, inhabitants of homes for the aged and men awaiting call-up for military service are included in this category.

†Median among those with any amount of activity (inter-quartile range).

Household activity was lowest for men, young participants, the Chinese, and those with higher SES (Figure 1). 92% of individuals reported at least some household activity. The largest difference in household activity was observed by gender with women both participating more in household activities and having a higher level of household activity (Table 3). For all measures of SES, we observed approximately double the level of household activity in those in the lowest as compared with those in the highest category of SES. The level of household activity of at least moderate intensity was much lower, but associations with sociodemographic characteristics were generally similar (Figure 2). However, the association between older age and more household activity largely disappeared.

### Total physical activity

Total physical activity was highest in Malays, intermediate in Asian Indians, and lowest in Chinese (Figure 1a). The lower activity in the Chinese participants was due to having less household and occupational activity rather than having less leisure time activity. Although a higher SES was associated with more leisure time physical activity, it was associated with less total physical activity (Figure 1b). This was due to the lower household, transport, and occupational activity in participants with a higher SES. The middle age group had the highest total physical activity as most people in this group were working (Figure 1c). Total physical activity was higher in men than women due to having higher leisure activity and occupational activity (Figure 1d). When considering only activities of moderate or higher intensity levels, total physical activity was still highest in Malays, the low SES group, the middle age group, and men (Figure 2).

The majority of the study population (71%) achieved the recommended level of activity of at least 150 minutes per week of moderately intense physical activity or 60 minutes of vigorous physical activity or a combination of both. The percentage of participants who did not meet the recommended level of activity was somewhat higher for women (31%), the youngest age group (18-39 years; 34%), and those with the lowest (lower primary education or less; 36%) or highest (university; 33%) education. The percentage of participants not meeting the physical activity recommendations also differed by work status: homemakers (26%) and the retired (29%) were the least likely to not to meet the recommendations. Ethnicity, household income and type of housing were not significantly associated with achieving the recommended level of activity (data not shown).

## Discussion

Identifying sociodemographic determinants of different types of physical activity is an important step in the planning of public health interventions. In this population-based study of 4750 Asian Indians, Malays, and Chinese men and women in Singapore, we evaluated the characteristics of individuals in relation to their energy expenditure through physical activity in different domains. We observed that household and occupational activity contributed most to total physical activity, whereas leisure and transportation related activities contributed much less. However, when only activities at least moderate intensity were considered, leisure time activity contributed most to total physical activity. Participants with a higher SES had more leisure time activity, but less occupation, transport and household activity resulting in lower overall physical activity. In addition, Chinese ethnicity was associated with lower levels of total physical activity than Malay and Asian Indian ethnicity. If only leisure time activity were considered, the wrong socio-demographic groups would be assumed to have the lowest physical activity leading to wrong priorities for physical activity interventions. In addition, non-leisure activities such as transportation-related activities are domains where intervention can provide more opportunities for individuals to achieve the recommended levels of activity.

When only activities of at least moderate intensity were considered, the association of gender, ethnicity, and SES with activity in different domains and all domains combined remained similar. When activity of all intensities was evaluated, the oldest age group had more leisure time and household activity than the youngest age group. However, these differences largely disappeared when only activity of at least moderate intensity was considered; suggesting that for older participants, a larger proportion of household and leisure time activity was of light intensity.

The findings in this study are largely consistent with those of other studies. Women were more active than men in household activities [33,34], whereas men were more likely to participate in leisure time and occupational activity than women [12,35,36]. Older age, unemployment and being retired were associated with more household activity [33]. A higher level of education and a higher income were associated with more leisure time activity [14,35,37,38], but less occupational activity [33,35]. Unlike previous studies that reported that homemakers were less physically active [39,40], our study showed that this group expanded more energy through total physical activity than the population working outside the home. Consistent with our findings, in a study conducted by Mein et al full time employment was associated with a lower number of people achieving the recommended physical activity level, whereas retirement was associated with higher total activity levels [41].

There are several strengths to our study. Firstly, this was a large study with a comprehensive assessment of all domains of physical activity in this population including different ethnicities using a locally validated questionnaire. This study included significant numbers of Malays and Asian Indians to improve precision of the assessments in these groups, and together with the Chinese, we were able to study three Asian ethnic groups which represent a large part of the population in Asia. Secondly, the use of different social economic indicators such as education, occupation, household income and type of housing enabled a more comprehensive assessment of the social economic status of the study population. In addition, the study was conducted in an urban setting in a multi-ethnic population in Singapore and may provide some important lessons for other countries in the region that are currently undergoing rapid urbanization.

We acknowledge that a limitation was that the physical activity in our study was based on self-report and likely to have been affected by measurement error. Our analyses mainly relied on the percentage of participants participating in an activity and the median level of activity, which may be less affected by outliers due to over-reporting of activity on the questionnaire than analyses based on means. In addition, we focused mainly on the comparison of the amount of physical activity between different groups rather than the estimation of absolute levels of physical activity. However, we cannot exclude the possibility that differences in measurement error by sociodemographic group may have affected our results.

Another limitation is that the calculation of physical activity values was based on the published MET values derived from non-Asian populations which may be higher than for Asians [42-44]. However, to our knowledge, comprehensive MET tables are not available for Asians. As all participants in this study were Asian this limitation may not have affected the comparison of different sociodemographic groups. Although there was a non-ideal response rate (51%), our previous work showed that responders were slightly but not greatly different to non-responders (data is available on request). It should also be noted that the energy expenditure estimation in this study was based on the commonly used assumption that 1 MET equals 1 kcal × kg body weight^-1 ^× h^-1^. However, Bryne et al demonstrated that this calculation could overestimate resting energy expenditure by 20% [45].

Most occupations in Singapore, as in other developed countries, do not involve much physical activity. Therefore, there is a need to consider opportunities to increase physical activity in other domains, for example encouraging more leisure time activities in the lower SES group and facilitating transportation activity for the whole population. Collaborations between the health sector, transportation, town and park planners and local government agencies can contribute to achieving these goals. Examples of potential interventions to increase physical activity include the development of walking trails that link smaller local parks, creating undulating areas and maintenance of care of the area [46]; providing good access to larger public open space with more attractive attributes [47]; and redesigning existing playing fields with public access for multiple users [48]. In addition, programmes that encourage incorporating walking to one's daily activity for example using the stairs instead of elevators, parking further away from their destinations, exiting public transport before their destination and providing a positive social environment such as walking with others could be ways of improving leisure and transportation activities [49].

## Conclusion

This study identifies different sociodemographic factors associated with physical activity patterns in transportation, household, leisure time and occupation. We found that low socioeconomic status was associated with transportation, household and occupational activity while high socioeconomic status was associated with leisure time activity. We also found different patterns in age groups and gender in different domains. It is important to consider activities in different domains and population subgroups prior to implementing public health intervention in order to target the right high-risk, physically inactive individuals. Our results suggest that various domains of activity may benefit from potential health interventions. This includes increasing levels of activity related to transportation by facilitating and improving infrastructure, promoting leisure time related activities in the younger and middle aged age groups, Chinese and lower SES group, encouraging more leisure related activities in women and more household activity in men. The end goal is to increase physical activity, improve quality of life and reduce the burden of chronic diseases.

## Competing interests

The authors declare that they have no competing interests.

## Authors' contributions

EST and JL designed the study and directed its implementation, including quality assurance and control. EEKN and EYHK helped conduct the literature review and data analysis and drafted the manuscript. AS helped in data analysis and interpretation. RMVD helped the interpretation of data and led writing of the manuscript. All authors read and approved the final manuscript.

## Pre-publication history

The pre-publication history for this paper can be accessed here:

http://www.biomedcentral.com/1471-2458/10/644/prepub
